# New Vision of Cell Walls in *Aspergillus fumigatus* from Solid-State NMR Spectroscopy

**DOI:** 10.3390/jof10030219

**Published:** 2024-03-16

**Authors:** Isha Gautam, Kalpana Singh, Malitha C. Dickwella Widanage, Jayasubba Reddy Yarava, Tuo Wang

**Affiliations:** Department of Chemistry, Michigan State University, East Lansing, MI 48824, USA; gautamis@msu.edu (I.G.); singhkal@msu.edu (K.S.); malithdw@magnet.fsu.edu (M.C.D.W.); yaravaja@msu.edu (J.R.Y.)

**Keywords:** cell wall, fungi, *Aspergillus*, solid-state NMR, carbohydrate, polysaccharide, chitin, glucan, antifungal

## Abstract

The fungal cell wall plays a critical role in regulating cellular integrity and communication, and serves as a frontline defense against stress. It is also a prime target for the development of antifungal agents. The cell wall is comprised of diverse polysaccharides and proteins and poses a challenging target for high-resolution structural characterization. Recently, the solid-state nuclear magnetic resonance (ssNMR) analysis of intact *Aspergillus fumigatus* cells has provided atomic-level insights into the structural polymorphism and functional assembly principles of carbohydrate components within the cell wall. This physical perspective, alongside structural information from biochemical assays, offers a renewed understanding of the cell wall as a highly complex and dynamic organelle. Here, we summarize key conceptual advancements in the structural elucidation of *A. fumigatus* mycelial and conidial cell walls and their responses to stressors. We also highlight underexplored areas and discuss the opportunities facilitated by technical advancements in ssNMR spectroscopy.

## 1. Introduction

**Figure 1:**
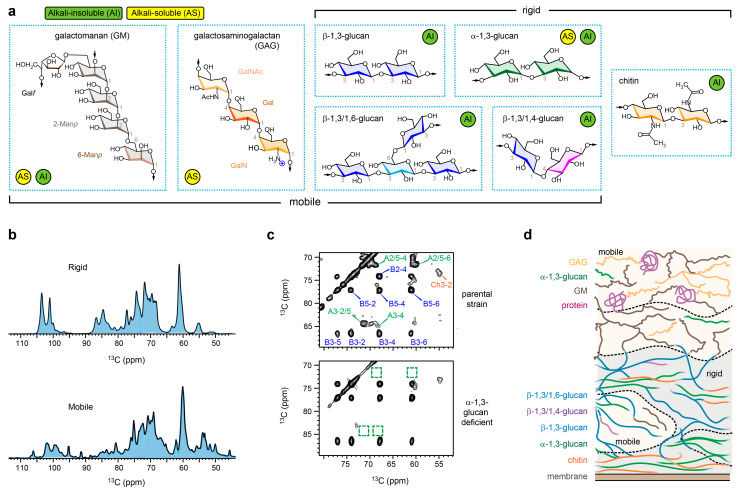
Polymer structure and dynamics in *A. fumigatus* mycelia viewed by solid-state NMR. (**a**) Simplified structural representation of key polysaccharides categorized based on their mobility (mobile or rigid) and their alkali solubility (alkali-soluble: AS; alkali-insoluble: AI), with the key linkage sites numbered. Sugar units with diverse linkages are color-coded for 3-Glc*p* (blue), 4-Gl*p* (magenta) and 3,6-Glc*p* (cyan) in β-glucans, 4-GlcNAc (orange) in chitin, and 3-Glc*p* in α-1,3-glucan (green). (**b**) Selective detection of molecules with distinct dynamics using intact *A. fumigatus* cells, with two spectra showing different signals. (**c**) Representative 2D ^13^C-^13^C correlation spectra, providing site-specific resolutions for discerning various carbon sites in cell wall polymers, where each dot represents correlations between two different carbons within a single molecule. The top spectrum of parental strain shows signals of chitin (Ch), β-1,3-glucan (B), and α-1,3-glucan (A). The bottom spectrum of α-glucan-deficient mutant lacks signals of α-1,3-glucan, as highlighted using dashed line boxes. For example, A3-4 represents the cross peak between carbons 3 and 4 in α-1,3-glucan, which becomes absent in the mutant. (**d**) Organization of biomacromolecules within the cell wall of *A. fumigatus mycelia*. Panels (**c**,**d**) adapted from Chakraborty et al. *Nat. Commun.* (2021) [10].

The fungal cell wall, an intricate and dynamic composite of polysaccharides and proteins positioned outside the plasma membrane, serves as the frontline in the interaction between fungi and their environment [1,2]. This organelle plays a pivotal role in governing fungal survival, facilitating cellular communication, and influencing both fungal virulence and the host’s immune response [2,3]. The carbohydrate components and their biosynthesis are crucial targets of antifungal agents, with recent successes being exemplified by the development of β-glucan inhibitors such as echinocandins and ibrexafungerp [4,5]. Our understanding primarily relied on chemical assays delineating the linkage and composition of carbohydrate components post-extraction and -isolation through enzymatic and chemical digestion (e.g., alkali), coupled with imaging techniques for molecular localization within the cell wall [6,7,8]. Within the cell wall of the widespread airborne pathogen *Aspergillus fumigatus*, the alkali-insoluble portion, exhibiting fibrillar structures, encompasses covalently linked β-glucans, chitin, and galactomannan [9] (Figure 1a). Additionally, a small proportion of α-1,3-glucan has been detected within the alkali-insoluble fraction of *A. fumigatus* cell walls [10]. The alkali-soluble fraction, characterized by amorphous molecules, contains galactomannan, galactosaminogalactan, and α-1,3-glucans [11,12].

In the last five years, solid-state nuclear magnetic resonance (ssNMR) has played a crucial role by offering distinctive insights into the physical characteristics, such as mobility and packing interactions, as well as the structural complexity of cell wall polysaccharides [13]. This complements the existing knowledge, contributing to a renewed understanding of the *A. fumigatus* cell wall [14,15]. The characterization of *A. fumigatus* was informed by ssNMR studies conducted on various fungal species, particularly *Cryptococcus* species, pioneered by Stark, Casadevall, and colleagues [16,17,18], along with prior investigations into diverse cell wall systems, including plant cell walls led by Hong and colleagues [19,20], and studies of bacterial and algal extracellular matrices [21,22,23,24,25]. While only a few high-resolution ssNMR studies on *Aspergillus* cell walls [10,26,27,28,29,30] and biofilms [31,32] have been conducted thus far, the methodologies and comprehension of spectroscopic observables are established. We are now poised to utilize these methods in order to investigate numerous unresolved questions pertaining to cell wall structure and remodeling, offering insights into fungal biology, environmental adaptation, and antifungal resistance. Hence, this review aims to highlight recent advancements in our understanding of *A. fumigatus* cell walls, as well as to identify key challenges and unanswered questions that warrant further exploration.

## 2. Indirect Assessment of Cell Wall Organization in *A. fumigatus* Mycelia

Mapping the arrangement of polymers within complex and heterogeneous polymer composites, such as fungal cell walls, poses technical challenges. SsNMR offers a means of indirectly deducing such pertinent information as demonstrated in recent investigations of *A. fumigatus* cell walls [10,27]. This is achieved by integrating information on polymer dynamics, water interactions, and physical intermolecular arrangements, employing techniques previously developed for studying the structural properties of proteins and polymers [33,34,35,36]. While maintaining the integrity of the fungal cell during ssNMR measurements, distinct parts of the cell, the cell wall, and their constituent polymers can be selectively examined (Figure 1b). This selectivity is achieved by leveraging differences in rigidity or, more precisely, the physical parameters determined by molecular rigidity. For instance, rigid molecules often display relatively slow relaxation rates accompanied by strong dipolar couplings. Consequently, these molecules can be selectively detected utilizing relaxation filters or dipolar-based polarization techniques in either one-dimensional or multidimensional formats, with the latter providing enhanced spectral resolution (Figure 1c) [37].

In the mycelia of *A. fumigatus*, the rigid scaffolds feature the anticipated chitin microfibrils, but, unexpectedly, they also include α-1,3-glucan [26]. These α-1,3-glucans exhibit intricate sub-nanometer packing with chitin, suggesting their deposition on chitin microfibril surfaces or their entrapment between multiple microfibrils. Consequently, these α-1,3-glucans are spatially confined and undergo rigidification (Figure 1d). Hydration data validated this structural concept, revealing that the densely packed cores of α-1,3-glucan and chitin exhibit limited water retention due to restricted accessibility to water molecules [26].

It should be noted that the interactions observed in current ssNMR studies of fungal cell walls are non-covalent physical contacts occurring at the sub-nanometer scale between two molecules. This distinction is crucial when contrasting them with the covalent bonding patterns of polysaccharides, which were determined through the chemical and spectroscopic analyses of carbohydrate components extracted from the fungal cell wall [6,8,9].

The prevailing notion has involved β-glucans as the primary crosslinking polysaccharide, connecting galactomannan and chitin in *A. fumigatus* cell walls [6,9,38]. However, ssNMR data demonstrates that the number of observed interactions between β-glucan and chitin is lower than those involving α-1,3-glucan and chitin [26]. Additionally, the ssNMR data reveals a dual distribution of β-glucans, present in both rigid and mobile phases within the cell walls of *A. fumigatus* hyphae [26]. Instead, the revised model proposes that, although certain β-glucans are covalently attached to chitin microfibril terminals, a significant portion does not fold onto the chitin surface. Instead, they maintain an extended structure, projecting into the open space, and therefore contributing to the formation of a matrix that regulates water activity and porosity within the cell walls (Figure 1d). Remarkably, galactomannan, a crucial polysaccharide within the chemically characterized galactomannan-β-glucan-chitin core, was observed to exhibit high mobility. Consequently, this remains associated with structural proteins and galactosaminogalactan, residing predominantly on the surface and mobile layer of the cell wall [10].

## 3. Morphotype Transition in *A. fumigatus* Conidia

The structural organization of the *A. fumigatus* cell wall underwent significant rearrangement during the conidial morphotype transition, primarily influenced by compositional changes in α- and β-glucans. Recent findings by Loquet, Aimanianda, and colleagues indicated that the molar ratio between β-1,3-glucan and α-1,3-glucan in the rigid phase was approximately 3:1 in dormant conidia, and this ratio changed to almost 1:1 in swollen conidia, and then restored to around 3:1 in germinating conidia [27]. Throughout these transitions, the chitin content remained constant, with 12% of rigid carbohydrates in the cell wall. Sequential observations revealed a stiffening of the cell wall polysaccharide network and an increase in the water retention of glucans, transitioning from the dormant state to swollen conidia and then to germinating conidia [27]. These changes are indicative of intricate remodeling within the *A. fumigatus* cell walls. In dormant conidia, α-1,3-glucan and β-1,3-glucan are localized in the inner cell wall and shielded from the external environment by RodA rodlets. Swelling leads to the disruption of the RodA layer and melanin, facilitating higher water accessibility to both α- and β-glucans [27]. Under germinating conditions, the appearance of galactosaminogalactan in the mobile phase and the embedding of chitin into the inner layer of cell walls were also found crucial [27].

Irrespective of the conidia’s state, α-1,3-glucan consistently exhibits superior hydration within the rigid segment, surpassing both chitin and β-glucans [27]. This stands in contrast to the findings in mycelial cell walls, where α-1,3-glucan consistently appears as the most dehydrated and rigid molecule [10]. The discrepancy indicates that the association of α-glucan and chitin, and the subsequent stiffening of α-glucan, represents a distinctive structural characteristic during mycelial development. This also serves as molecular-level evidence of fungi’s remarkable structural dynamics, illustrating how the same molecule can engage in diverse roles within the cell wall architecture as needed.

## 4. Structural Complexity of Invisible Polysaccharides in Chemical Assays

The first novel structural concept posits that structural complexity exists not only at the level of chemical diversity, as characterized by linkage analysis, but also at a more localized level, encompassing factors such as conformation (e.g., torsional angles) and hydrogen bonding. Such structural complexity observed in the native cell evident within native cellular environments greatly exceeds that found in extracted or dissolved molecules, where conformational distribution is diminished or even absent. In the dissolved state, such as in solution NMR, the rapid molecular tumbling averages out the conformational diversity of molecules, resulting in the same set of signals for chemically identical molecules. In their native physical state within the cell, conformational diversity is observable in solid-state NMR as peak multiplicity. As a result, solution NMR chemical shifts typically exhibit strong correlation with solid-state NMR values for highly dynamic matrix components, such as pectin in plant primary cell walls [39] and the three-fold xylan in secondary plant cell walls [40]. The same is not true, however, for rigid and fibrillar components, such as chitin in fungi and cellulose in plants [29,41]. A more in-depth exploration of the complexity of the structure and chemical shifts observed in cellular carbohydrates can be found in a recent review [42], and the NMR fingerprint of fungal carbohydrate can be accessed here [43].

**Figure 2:**
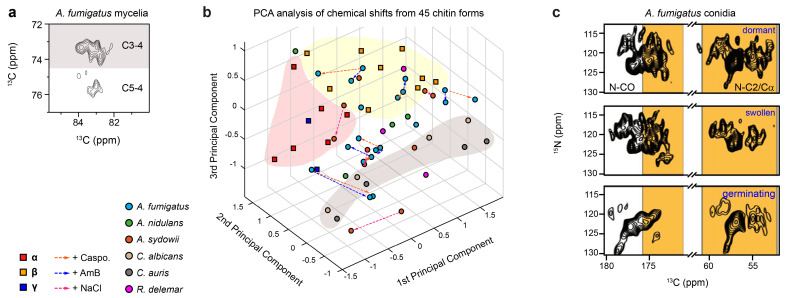
Structural polymorphism of chitin in *A. fumigatus* mycelia and conidia. (**a**) Representative spectral regions of chitin C3-C4 and C5-C4 correlations in *A. fumigatus* mycelia. Peak multiplicity is evident especially for the C3-C4 cross peaks, where each signal represents a unique conformer. (**b**) PCA analysis of 45 chitin forms from six fungal species, and comparisons with the literature data from model allomorphs (α, β, or γ-chitin). (**c**) Representative 2D ^15^N-^13^C correlation spectra of *A. fumigatus* conidia (top: dormant; middle: swollen; bottom: germinating), showing peak multiplicity for expected regions (yellow) of chitin signals. Panels (**a**,**b**) adapted from Fernando et al. *Front. Mol. Biosci.* (2021) [29]. Panel (**c**) modified from Lamon et al. *Proc. Natl. Acad. Sci. USA* (2023) [27].

Using chitin as an example, this chemically simple polymer, composed of β-1,4-linked N-acetylglucosamine (GlcNAc) units, was traditionally perceived as being uniform. However, ssNMR spectroscopy revealed 45 distinct chitin forms (each with a distinct conformation or variations in hydrogen bonds) in various fungal samples, including the mycelia of *Aspergillus* (*A. fumigatus*, *A. nidulans*, and *A. sydowii*) and *Rhizopus* species, as well as *Candida* cells (Figure 2a,b) [29]. In the case of the *A. fumigatus* mycelium alone, six clearly identified chitin forms featuring well-defined signals, along with two minor forms with ambiguous carbon sites, were resolved (Figure 2c) [26,29]. Consistently, a separate investigation of *A. fumigatus* conidia also revealed the presence of 5–10 distinct chitin subtypes (Figure 2c) [27]. Each form corresponds to a unique set of chemical shifts for carbon sites, reflecting variations in local structures.

Principal component analysis (PCA) of chemical shift data derived from 45 chitin forms present in fungal cell walls, along with 17 model allomorphs determined using isolated and purified materials, revealed notable structural differences [29]. This suggests that macromolecules within cellular contexts do not adopt exact structures, as observed in their purified forms. Notably, fungal chitin exhibited a closer alignment with the α-allomorph y antiparallel chitin packing, contrasting the β-allomorph characterized by parallel packing. Furthermore, changes in chitin structure were observed following antifungal treatments with amphotericin B (AmB) and caspofungin, as well as exposure to high salt conditions [29].

This observed structural heterogeneity has not been fully correlated with our current understanding of cell wall biosynthesis complexity. *A. fumigatus* possesses eight chitin synthase (CHS) genes, distributed across the structurally distinct families 1 and 2, with evidence from functional genomics suggesting cooperative interactions between these gene families [44]. These genes comprise four members in Family 1 (CHSA in Class I, CHSB in Class II, and CHSC and CHSG in Class III) and four members in Family 2 (CSMA in Class V, CSMB in Class VII, CHSF in Class IV, and CHSD in Class VI) [44,45]. In *C. albicans*, individual CHS genes have been linked to distinct chitin microfibrils and their specific localizations in the cell wall [46]. Therefore, it is also of great interest to investigate whether any of the NMR-identified chitin forms in *A. fumigatus* are essential for fungal growth, and if they are directly associated with specific CHS gene types or particular morphologies of chitin microfibrils.

Analogous complexity was also identified in α-1,3-glucan, particularly in *A. fumigatus* mycelia exposed to caspofungin [47,48]. This exposure led to an augmentation in the abundance of two minor forms of α-1,3-glucans [47], showing distinct chemical shifts at carbon 3, the glycosidic linkage site, particularly when compared to the predominant form found in wild-type *A. fumigatus* and its mutant strains [10], thus suggesting variations in their helical screw conformations. Similarly, three distinct types of α-1,3-glucan signals were also identified in the dormant conidia of *A. fumigatus* [27]. However, the potential correlation between these structural variations and the three α-1,3-glucan synthase (AGS) genes present in *A. fumigatus* remains unclear [49].

β-glucans are recognized for their intricate linkage patterns, e.g., branched β-1,3/1,6-glucan, linear β-1,3-glucan, and terminal β-1,3/1,4-glucan in *A. fumigatus* (Figure 1a) [6]. The diversity in linkages was primarily characterized using chemical analysis, and is presently distinguishable based on distinct ssNMR chemical shifts. As of now, there has been no observed conformational polymorphism in β-glucans within *A. fumigatus* mycelia or conidia [10,27]. This absence is unexpected for any biopolymers in native and heterogeneous biomaterials, where diverse conformations are typically anticipated and observable via NMR, particularly when interacting with rigid molecules such as chitin. This is possibly linked to the absence of spatial constraints for the majority of β-glucans, which enables them to undergo motions that render conformational distributions undetectable in ssNMR spectra. This hypothesis finds support in the substantial broadening of β-glucan signals when exposed to cryogenic temperatures (~100 K) during dynamic nuclear polarization (DNP) measurements [30]. This broadening of β-glucan peaks is ascribed to the restriction of motion and the entrapment of all conformations at low temperatures, while chitin partially maintains its linewidth, owing to its crystalline nature.

## 5. Tracking *Aspergillus*’ Structural Responses to Stress

Recently, we have assessed the structural implications on the *A. fumigatus* cell wall in response to compensatory reactions undertaken by fungi to address internal stressors. This investigation involved comparing the parental strain of *A. fumigatus* with four mutants, each lacking a specific polysaccharide in the cell wall—namely chitin, α-1,3-glucan, galactomannan, or galactosaminogalactan [10]. Common changes observed in all mutants included a decrease in cell wall thickness, an increase in polymer rigidity, and a decline in water accessibility. The disrupted cell wall biosynthesis likely contributed to the reduced thickness, while the latter two changes appear to be strategies employed to maintain structural integrity in response to the absence of specific polysaccharides. Interestingly, upon the removal of any of these components, *A. fumigatus* exhibited a complete reconfiguration of the biosynthesis of the remaining carbohydrate components [10]. This contrasts with plants, where a deficiency in the biosynthesis of a single carbohydrate may not significantly affect the content of the other cell wall carbohydrates.

The noteworthy structural dynamics observed in fungi, particularly the ability to swap the role of an individual carbohydrate with another, necessitates a heightened level of caution in the utilization of mutants to assess the structural function of cell wall polysaccharides. While a mutant displaying a phenotype change, such as growth defects, serves as evidence of the role of a specific polysaccharide, the absence of defects does not necessarily imply that the polysaccharide is nonessential in the wild-type cell wall. Instead, it indicates that the function of the polysaccharide can be fulfilled by other components due to the inherent structural flexibility of fungi. This phenomenon is exemplified by α-1,3-glucan, which stabilizes the rigid core of the *A. fumigatus* mycelial cell wall via its interaction with chitin [10,26]. Despite this crucial role, the AGS mutants lacking α-1,3-glucan did not exhibit growth defects [50,51]. Under α-1,3-glucan depletion, β-glucan synthesis increased, and chitin microfibrils became tightly packed in order to fortify the cell wall for survival [10].

Two additional investigations were carried out to assess how *Aspergillus* cell walls respond to external stressors, including changes in osmotic pressure and exposure to antifungal agents [28,47]. The initial study focused predominantly on a distinct *Aspergillus* species, *A. sydowii*, which is recognized as a model halophile which is capable of thriving in high-salt conditions [28]. Although *A. sydowii* shares strikingly similar NMR fingerprints with *A. fumigatus*, a notable distinction lies in the low content of α-1,3-glucan and the prevalence of chitosan in *A. sydowii*, though the former partially reappears when transitioning from low-salt to high-salt conditions. *A. sydowii*’s response to osmotic pressure induced by a hypersaline environment was found to be multifaceted [28]. The fungus demonstrated an enhancement in chitin biosynthesis while concurrently reducing β-glucan synthesis, resulting in the production of more rigid, thicker, and less permeable cell walls. The cell surface also exhibited an increased positive charge, which is attributed to the heightened content of galactosamine GalN, a cationic sugar residue of galactosaminogalactan (see chemical structure in Figure 1a), which facilitated enhanced surface adherence and intermycelial adhesion, thus presenting adaptive strategies to counteract challenging environments.

SsNMR has also been used to examine the restructuring of *A. fumigatus* cell walls when exposed to caspofungin treatment [47], revealing a strategy that bears resemblance to the approach utilized by *A. sydowii* in coping with hypersaline conditions. When β-1,3-glucan was largely depleted from the cell wall via caspofungin inhibition, *A. fumigatus* exhibits the enhanced production of chitin, thus augmenting cell wall rigidity and reducing water permeability. Additionally, the fungus relies on alternative carbohydrates, predominantly chitosan and novel forms of α-glucans, as crucial buffering molecules to uphold cell wall structural integrity in the absence of β-1,3-glucan, as well as to generate thicker cell walls [47]. These strategies seem to represent consistent adaptations across various *Aspergillus* species to counteract unfavorable environments; nevertheless, further investigations are essential to confirm these findings and to comprehend the structural mechanisms underpinning caspofungin paradoxical growth [52,53,54].

Essentially, ssNMR can serve as a pivotal tool for elucidating how fungi adapt to both antifungal treatments and diverse environmental challenges, which aligns with the pursuit of two of the five major unresolved questions concerning the fungal cell surface, as highlighted in a recent perspective article [55]. It is also noteworthy that recent ssNMR studies by Rienstra, Burke, and colleagues have determined the high-resolution structure of amphotericin B sterol sponges that encapsulate cholesterol and ergosterol [56,57]. This discovery served as the structural basis for the development of a new polyene with high antifungal potency against *Aspergillus* strains, but with low toxicity against human renal cells [56]. We anticipate that structural findings on *A. fumigatus* cell walls provided by ssNMR will also elucidate the mechanisms behind the inefficacy and resistance observed with current antifungals, thereby facilitating the development of improved wall-targeting antifungals.

## 6. Ambiguity in Assessing Protein and Lipid Components

Signals from carbohydrates, proteins, and lipids coexist in most solid-state NMR spectra, yet lipids and proteins are often understudied in the context of fungal cell walls due to the complexity of discerning their specific locations within the cell. The lipid and protein signals observed could stem from diverse origins, including those associated with cell walls (e.g., glycosylphosphatidylinositol-anchored proteins) [58,59], rodlets [60,61], plasma membranes, membrane proteins, and intracellular organelles. Additional chemical methodologies, such as cell wall isolation or carbohydrate extraction, are imperative for identifying lipids and proteins associated with the cell wall. For example, studies on *Cryptococcus* revealed that extracted melanized cell walls were associated with lipids [17,62], and subsequent spectral editing approaches on whole cells identified three key lipids in the cell: triglycerides, sterol esters, and polyisoprenoids [63]. Investigations into *A. fumigatus* mycelia demonstrated signals in alkali-insoluble fractions, indicating the covalent associations of polysaccharides with hydrophobic amino acids, such as valine [10]. The valine signals disappeared in mutants which lacked galactomannan and galactosaminogalactan [10], revealing the role of these two carbohydrates in stabilizing cell wall proteins. Further investigations, however, are needed to isolate and identify these protein–carbohydrate complexes. In addition, signals which are suggestive of triacylglycerol were observed in *A. fumigatus* conidia cells, but their specific association with cell wall polysaccharides remains to be elucidated [27]. Overall, careful consideration is warranted when interpreting the ssNMR signals of lipids and proteins, given their broad distribution across various cellular compartments.

## 7. Perspectives

The application of solid-state NMR techniques, coupled with biochemical findings, has enhanced our understanding of the physical characteristics of seven primary polysaccharides and their unaltered arrangement within native *A. fumigatus* cell walls. Chitin imparts rigidity to the cell wall, allowing molecules associated with it to attain partial rigidity, including α-1,3-glucan and β-glucans [26]. β- and α-glucans constitute the flexible matrix within the cell wall, with β-glucans facilitating water binding in *A. fumigatus* mycelia and α-glucans regulating water activity in *A. fumigatus* conidial cell walls [10,26,27]. The structural diversity of β-glucans, including linear β-1,3-glucan, terminal β-1,3/1,4-glucan, and branched β-1,3/1,6-glucan, is vital for maintaining molecular complexity and matrix formation. The latter two forms of glucans persist even in the absence of β-1,3-glucan; this is due to caspofungin treatment and interactions with other polysaccharides (α-1,3-glucan and chitin) to reinforce cell wall stability [47]. Although the level of chitin deacetylation is typically low in *A. fumigatus* mycelial and conidia samples [10,27], chitosan content may increase in response to stress [28,47]. Galactomannan is covalently linked to β-1,3-glucans, which are sometimes further cross-linked to chitin [9], but are the most mobile components within this polysaccharide complex [10]. Instead, it predominantly protrudes into the outer layer, along with galactomannan, in order to bolster the structural proteins within the cell wall [10]. Galactomannan plays a crucial role in preserving the charge of the cell wall surface, particularly for the mycelial cell wall and germinating conidia [10,27]. It also acts as a masking molecule, concealing β-glucans that are embedded deeper within the structure.

Certain structural motifs, such as α-1,4-glucose residues found in α-glucan (a minor component) [6] and melanin, the aromatic-rich pigment closely associated with *A. fumigatus* cell walls, have not yet been evaluated using solid-state NMR (ssNMR). In addition, the galactomannan in *A. fumigatus* possesses a complex chemical structure, featuring a linear backbone composed of α-1,2-linked mannotetraose repeating units, bridged via α-1,6-linkage [64,65]. Some of the α-1,2-linked mannose residues of the backbone are further branched at C-6 and C-3 positions by galactofuran sidechains consisting of several (on average, 4 to 5) β-1,5-galactofuranose units [65]. This structural elucidation was achieved via the solution NMR analysis of extracted galactomannan. Solid-state NMR has only resolved signals from the predominant structural units, such as the repeating β-1,5-galactofuranoses in the side chains and the α-1,2-linked and 1,6-linked mannoses in the backbone, while the branching sites remain unresolved. The functional role of the galactofuranose sidechains of galactomannan remains unclear as well. To elucidate the functional principles of these biomolecules, a combination of ssNMR with carefully selected mutants may be necessary.

Furthermore, some structural insights into *Aspergillu*s cell walls derived from biochemical and genomic data do not entirely correspond with ssNMR observations. A recent study encountered challenges in correlating transcriptomics data, reflecting the expression levels of various cell wall-related enzymes (such as synthases, transferases, glucanases, chitinases, hydrolases, etc.), with the final cell wall structure observed via NMR [28,66]. This suggests that the construction and modification of the cell wall are more intricate than we currently understand, and additional, unexplored pathways may also influence the resulting cell wall structure. These aspects permit further investigation.

Due to its biophysical nature, in-depth ssNMR analysis often demands extensive time, typically between one and several years. Consequently, its effectiveness in analyzing the chemical structure of carbohydrates is relatively restricted, instead favoring the utilization of solution NMR and chemical approaches. The primary advantage of ssNMR lies in in its ability to examine intact cells and its unique capability to provide insights into the atomic- and molecular-level interactions, dynamics, and other physical properties of cell wall macromolecules. Additional challenges are presented by the demanding isotopic enrichment (e.g., with ^13^C and ^15^N). These factors may restrict sample preparation conditions and the quantity of samples that can be analyzed, often presenting challenges in accurately aligning the sample conditions with those analyzed in other assays, thus potentially resulting in discrepancies. One approach to address these challenges is the utilization of ^1^H detection methods [67,68], recently applied to ^13^C/^15^N-labeled fungal materials, as demonstrated by Baldus, Wösten, and colleagues in *S. commune* [69,70,71]. Once we become thoroughly acquainted with the carbohydrate NMR fingerprints, these methods could be directly applied to unlabeled fungal materials since ^1^H is present at 99.99% natural isotopic abundance. Additionally, sensitivity-enhancing techniques like DNP have been employed with success on various unlabeled biomaterials [72,73], including *Aspergillus* mycelia and conidia [30]. These technical advancements will streamline and expedite the assessment of cell walls in *Aspergillus* and numerous other fungal species, across diverse biological, environmental, industrial, and biomedical contexts.

## Data Availability

No new data were created or analyzed in this study. Data sharing is not applicable to this article.
